# Functional Assessment of Residues in the Amino- and Carboxyl-Termini of Crustacean Hyperglycemic Hormone (CHH) in the Mud Crab *Scylla olivacea* Using Point-Mutated Peptides

**DOI:** 10.1371/journal.pone.0134983

**Published:** 2015-08-11

**Authors:** Chun-Jing Liu, Shiau-Shan Huang, Jean-Yves Toullec, Cheng-Yen Chang, Yun-Ru Chen, Wen-San Huang, Chi-Ying Lee

**Affiliations:** 1 Department of Biology, National Changhua University of Education, Changhua, Taiwan; 2 Sorbonne Universités, UPMC Université Paris 06, UMR 7144 CNRS, Equipe ABICE, Station Biologique de Roscoff, Roscoff, France; 3 CNRS, UMR 7144, Adaptation et Diversité en Milieu Marin, Station Biologique de Roscoff, Roscoff, France; 4 Institute of Bioinformatics and Structural Biology, National Tsing Hua University, Hsinchu, Taiwan; 5 Department of Biology, National Museum of Natural Science, Taichung, Taiwan; 6 Department of Life Sciences, National Chung Hsing University, Taichung, Taiwan; International Centre for Genetic Engineering and Biotechnology, ITALY

## Abstract

To assess functional importance of the residues in the amino- and carboxyl-termini of crustacean hyperglycemic hormone in the mud crab *Scylla olivacea* (Sco-CHH), both wild-type and point-mutated CHH peptides were produced with an amidated C-terminal end. Spectral analyses of circular dichroism, chromatographic retention time, and mass spectrometric analysis of the recombinant peptides indicate that they were close in conformation to native CHH and were produced with the intended substitutions. The recombinant peptides were subsequently used for an *in vivo* hyperglycemic assay. Two mutants (R13A and I69A rSco-CHH) completely lacked hyperglycemic activity, with temporal profiles similar to that of vehicle control. Temporal profiles of hyperglycemic responses elicited by 4 mutants (I2A, F3A, D12A, and D60A Sco-CHH) were different from that elicited by wild-type Sco-CHH; I2A was unique in that it exhibited significantly higher hyperglycemic activity, whereas the remaining 3 mutants showed lower activity. Four mutants (D4A, Q51A, E54A, and V72A rSco-CHH) elicited hyperglycemic responses with temporal profiles similar to those evoked by wild-type Sco-CHH. In contrast, the glycine-extended version of V72A rSco-CHH (V72A rSco-CHH-Gly) completely lost hyperglycemic activity. By comparing our study with previous ones of ion-transport peptide (ITP) and molt-inhibiting hormone (MIH) using deleted or point-mutated mutants, detail discussion is made regarding functionally important residues that are shared by both CHH and ITP (members of Group I of the CHH family), and those that discriminate CHH from ITP, and Group-I from Group-II peptides. Conclusions summarized in the present study provide insights into understanding of how functional diversification occurred within a peptide family of multifunctional members.

## Introduction

Crustacean hyperglycemic hormone (CHH) is a peptide hormone originally identified in a crustacean neurosecretory complex, the X-organ/sinus gland complex (XO/SG complex), located within the eyestalks of decapod crustaceans (see [1,2]). CHH belongs to a family of peptide hormones, the CHH family [2,3,4]; the family also includes molt-inhibiting hormone (MIH), vitellogenesis-inhibiting hormone (VIH), and mandibular organ-inhibiting hormone (MOIH), and insect ion-transport peptide (ITP) [4,5,6]. Recently, existence of CHH-family peptides has been expanded beyond arthropods to ecdysozoans (*e*. *g*., [7,8,9]).

Combined data from studies of CHH found that it plays regulatory roles in the main pathways of carbohydrate metabolism (see [10]) and is considered a stress hormone that elicits stress-induced hyperglycemia [11,12,13,14,15]. Existing data suggest that several other physiological processes, *e*. *g*., molting, osmoregulation, and reproduction [16,17,18,19,20,21,22,23], may also be regulated by CHH. In addition, recent studies discovered expression of CHHs, some of which with unique sequences not previously reported, in circulating hemocytes [24,25]; it was also demonstrated that *in vivo* injection of recombinant CHH significantly increased pathogen clearance ability and survival rate of pathogen-infected shrimps [26]. The combined results suggest that novel function(s) of CHH have yet to be characterized.

Sequence analysis of CHHs isolated from various decapod crustaceans shows they are peptides of 72–73 amino acid residues, with 3 disulfide bridges formed by 6 highly position-conserved cysteine residues, which is a signature characteristic for the CHH family [2]. Peptide sequencing analyses indicated that CHH is N- and C-terminally blocked; the C-terminal amide was found critical for its hyperglycemic activity [16,27,28,29]. Molecular characterizations of CHH precursors indicated that the precursor consists of a signal peptide, a CHH precursor-related peptide (CPRP), and a mature CHH peptide. Based on this and other sequence characteristics, it was proposed that CHH peptides, along with ITP, be categorized as members of Group I of the CHH family, whereas MIH, VIH/GIH, and MOIH (the precursors of which lack CPRP) be categorized as members of Group II [4,6,30]. Motif analysis of CHH-family peptides revealed 2 common motifs (Motif A2 and A3) in the center of the peptide shared by both Group I and II, which could be distinguished by a group-specific N-terminal motif (A1 and A1′ for Group I and II, respectively) and, to a lesser extent, by 2 C-terminal motifs (A4, A5 and A4′, A5′ for Group I and II, respectively) [30].

Although it is known that CHH-family peptides exhibit distinct functions for each constituting member, respectively in carbohydrate metabolism, molt-inhibition (suppression of ecdysteroid synthesis and release), reproduction (inhibition of vitellogenesis; inhibition of synthesis and release of methyl farnesoate), and ion and water balance, early studies have demonstrated that some members, especially CHH, express activity ascribed to other members as mentioned above (see also [31,32] for reviews). Thus, it is particularly interesting to understand, at molecular level, how each distinct function is structurally defined and how certain members appear multifunctional, expressing biological activities often shared by other family members. It is rather surprising that no systematic study of structure-function relationship has been carried out for CHH, the prototypical member of the CHH family. Among CHH-family peptides, functional analyses using point-mutated peptides have been previously performed mainly for *Schistocerca gregaris* ITP (Scg-ITP) and *Marsupenaeus japonicas* MIH (Maj-MIH) [33,34,35]. In addition, Maj-MIH is the first and thus far the only CHH-family peptides whose tertiary structure has been resolved [36]. Results of the Maj-MIH studies suggested that functional important sites are located in an N-terminal α-helix (α1) and the C-terminal end for MIH [27,33]. For CHH, it has been shown that substitution of serine for cysteine^26^ and cysteine^43^, 2 of the 6 conserved cysteine residues, and substitution for 2 other residues (aspartate^12^ and asparagine^28^) effectively reduced its hyperglycemic activity in *Astacus leptodactylus*; experiments using deleted mutants showed that a Δ5 mutant (that lacks Motif A5 and the remaining C-terminal residue) detained hyperglycemic activity at levels not significantly different from that of wild-type [37], a finding contradictory to those of previous studies that the C-terminal end is functionally important [16,27,28,29]. In the present study, a more comprehensive screening of CHH using point-mutated and C-terminally amidated peptides of the mud crab *Scylla olivacea* CHH (Sco-CHH) was performed, with several mutation sites shared by previous studies of Maj-MIH and Scg-ITP. Comparing the results of these studies should give insights into the functional relevance of the motifs to which functionally critical residues belong and more importantly into how functional diversification occurred between subgroups of the CHH family through changes in critical residues.

## Materials and Methods

### Animals and ethics statement

Adult mud crabs *Scylla olivacea* were provided by a local supplier, transported to the laboratory, and reared in tanks containing artificial seawater (20‰ salinity), which was continuously aerated and circulated through a bio-filter, at a 24–26°C water temperature and under a 12L:12D photoperiod regimen [16]. Animals were staged according to accepted criteria [38] and only adults (range of carapace width: 8–9 cm) in intermolt stage were used in the present study.

Experimental protocols involving animals employed by the present study were approved by the review committee of National Changhua University of Education (Permit number: NCUE-94320003), in full accordance with the recommendations (Guidelines for Management and Use of Experimental Animals) set by the Council of Agriculture, Taiwan. No animal was sacrificed due to implementation of the protocols. Animals were returned to the rearing tanks after experiments were completed.

### Construction of plasmids encoding alanine-substituted Sco-CHH-Gly

**Fig 1:**
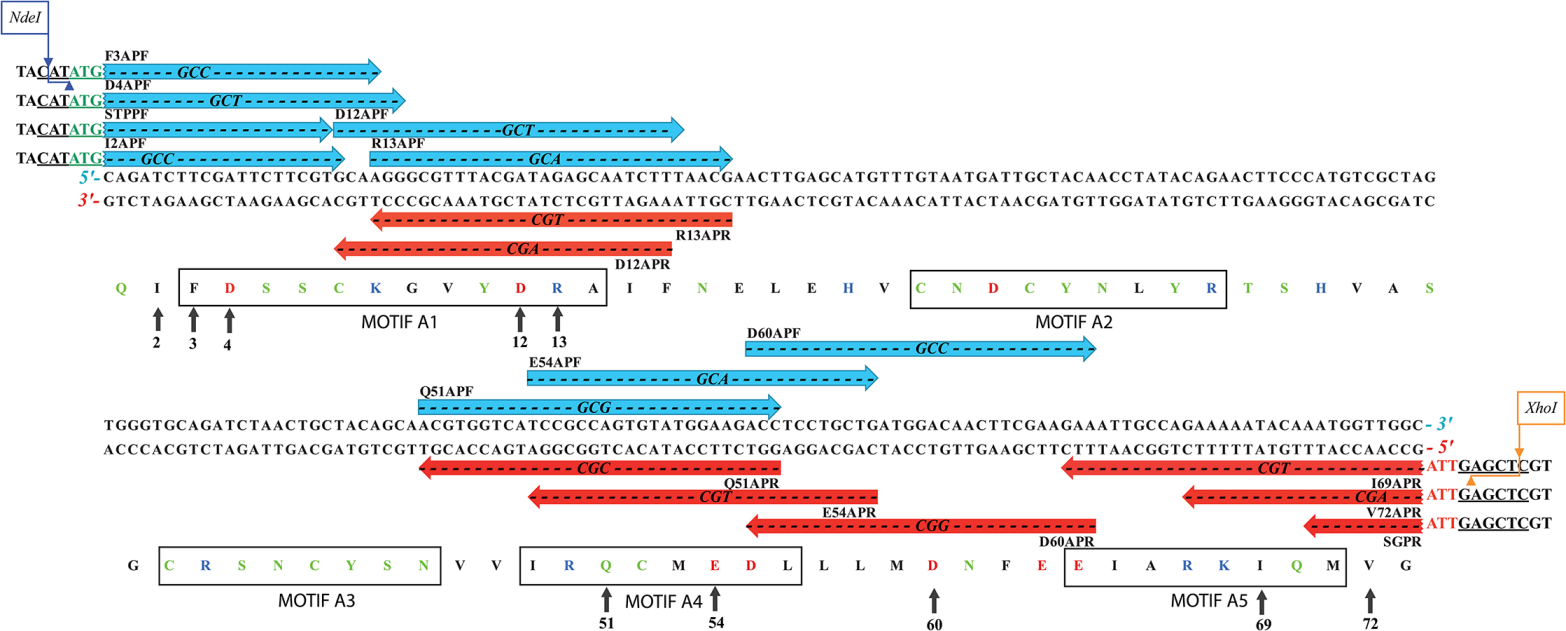
Location and sequence of the PCR primers used in the present study. DNA sequences (upper: sense strand; lower: anti-sense strand) encoding a glycine-extended version of the mature Sco-CHH (accession No. AAQ75760) are given. Forward (blue) and reverse (red) primers are placed above the sense strand and below the anti-sense strand, respectively. Nucleotide sequences of the primers identical to those of the Sco-CHH sequence are indicated by bold dash line (- - - -), with the alanine-encoding codons in the forward primers (GCC, GCT, GCA, or GCG) and in the reverse primers (GGC, AGC, or TGC) shown in italics. *NdeI* recognition sequence (CATATG) containing a methionine-encoding start codon (ATG, shown in green) was included in the forward primers STPPF, I2APF, F3APF, D4APF; *XhoI* recognition sequence (CTCGAG) was included in the reverse primers SGPR, I69APR, V72APR, followed by a stop codon (TTA shown in red) and a glycine-encoding codon GCC. Amino acid sequence of the glycine-extended Sco-CHH is also shown. CHH motifs as defined by Lacombe et al. [30] are boxed. Residues for which alanine was substituted in the present study are pointed by arrows and the residue numbers given.

To amplify DNA fragments each encoding an aniline-substituted and glycine-extended CHH, a recombinant plasmid, Sco-CHH-Gly/pET-22b(+) with an insert encoding the *Scylla olivacea* glycine-extended CHH (Sco-CHH-Gly) [16], was used as the template in polymerase chain reaction (PCR), except where noted otherwise. Sequence and location of PCR primers used are given and shown in Fig 1. For PCR amplification of the products encoding I2A, F3A, and D4A Sco-CHH-Gly, a forward primer (I2APF, F3APF, and D4APF, respectively) was paired with a common reverse primer SGPR; for PCR amplification of the products encoding I69A and V72A Sco-CHH-Gly, a common forward primer STPPF was paired with a reverse primer (I69APR and V72APR, respectively). In order for subsequent cloning into the expression vector, *NdeI* site sequence (CATATG containing a methionine-encoding start codon ATG) was included in the forward primers STPPF, I2APF, F3APF, D4APF; *XhoI* site sequence (CTCGAG) was included in the reverse primers SGPR, I69APR, V72APR, followed by a stop codon (TTA) and a glycine-encoding codon GCC (see Fig 1). With the exception of STPPF and SGPR, the forward primers contain an alanine-encoding codon (GCC, GCT, GCA, or GCG) and the reverse primers a reverse and complementary codon for alanine (GGC, AGC, or TGC) for introducing an alanine residue into the intended mutation sites (see Fig 1).

For amplification of the products encoding D12A, R13A, Q51A, E54A, and D60A Sco-CHH-Gly, two separate PCRs were first performed, both using Sco-CHH-Gly/pET-22b(+) as the template, with one reaction using a forward primer containing an alanine-encoding codon (D12APF, R13APF, Q51APF, E54APF, and D60APF, respectively) paired with a common reverse primer SGPR and the other reaction a common forward primer STPPF paired with a reverse primer containing an alanine-encoding codon (D12APR, R13APR, Q51APR, E54APR, and D60APR, respectively) (see Fig 1). The PCR products amplified by the 2 reactions were purified (Gel-M Gel Extraction System, Viogene), combined, and used as the templates for a third PCR reaction that used STPPF and SGPR as amplification primers.

All PCR reactions were performed using reagents supplied by a commercially available kit (GoTaq Flexi DNA polymerase, Promega) under the following parameters: an initial denaturation (5 min, 95°C), 35 cycles of denaturation (30 s, 95°C), annealing (30 s, 60°C—67°C depending on the primers used), and extension (30 s, 72°C), followed by a final extension (30 s, 72°C).

The amplified PCR products were individually cloned into an expression vector pET-22b(+) (Novagen) resulting in recombinant plasmids I2A, F3A, D4A, D12A, R13A, Q51A, E54A, D60A, I69A, and V72A Sco-CHH-Gly/pET-22b(+). The resulting recombinant plasmids were used to transform JM109 competent cells (ECOS, Yeastern Biotech Co., Ltd.). Colonies of the transformed cells were selectively grown on ampicillin-containing (100 μg/ml) LB (Luria-Bertani) agar plates and extracted for the recombinant plasmids (Mini Plus, Viogene) for confirmation of the DNA sequences using an ABI 3730 autosequencer.

### Production and refolding of recombinant wild-type and alanine-substituted Sco-CHH-Gly

The wild-type Sco-CHH-Gly and alanine-substituted Sco-CHH-Gly peptides were produced by an *Escherichia coli* expression system as described previously [16]. Briefly, the recombinant plasmids, Sco-CHH-Gly/pET-22b(+), and I2A, F3A, D4A, D12A, R13A, Q51A, E54A, D60A, I69A, V72A Sco-CHH-Gly/pET-22b(+), were used to transform competent One Shot BL21(DE3) *E*. *coli* cells (Invitrogen). The transformed cells were grown in ampicillin-containing (100 μg/ml) LB medium and induced by 1 mM isopropyl β-D-1-thiogalactopyranoside (IPTG); 4 h after induction, cells were harvested, disrupted, and centrifuged using established protocols as previously described [16]. Aliquots of total cell homogenate, supernatant, and pellet (dissolved in 6M guanidine-HCl) were analyzed for protein expression by tricine–sodium dodecyl sulphate–polyacrylamide gel electrophoresis (Tricine–SDS–PAGE) according to Schagger and Von Jagow [39].

The pellets, dissolved in 6M guanidine-HCl, were loaded onto a C_18_ Sep-Pak cartridge (WAT043345, Waters), which was eluted with 60% acetonitrile (ACN). The 60% ACN-eluted fractions were collected and lyophilized (CVE-200D, EYELA). The resulting dried materials were dissolved in ethylene glycol (0.8 mg/ml) and mixed (2:9 v/v) with a refolding buffer (250 mM Tris base, 5 mM cystine, 0.5 mM cysteine, 1.5 M urea) at 4°C for 20 h, after which the solution containing the refolded proteins was filtered through a 0.22-μm polyvinylidene fluoride (PVDF) membrane, injected onto a reversed phase column coupled to a HPLC system, and eluted using an elution gradient of ACN, as previously described [16]. The HPLC-purified recombinant peptides were separated by Tricine-SDS-PAGE, and the gels stained with Coomassie blue for protein visualization.

### Circular dichroism spectral analysis of recombinant wild-type and alanine-substituted Sco-CHH-Gly

Circular dichroism (CD) spectra were obtained using an AVIV 202 spectropolarimeter (Aviv Associates). The HPLC-purified glycine extended wild-type Sco-CHH (Sco-CHH-Gly), and glycine extended and alanine-substituted Sco-CHHs (I2A, F3A, D4A, D12A, R13A, Q51A, E54A, D60A, I69A, and V72A Sco-CHH-Gly) were individually dissolved in 10 mM PBS (150 mM NaCl, pH 6.8) at 20–30 μM; an aliquot (200 μl) of the samples was placed in a 1-mm cell and data collected from 260 nm to 200 nm in 0.5 nm increments at 25°C. Raw data were baseline corrected, smoothed, and transformed to obtain spectra in units of mean residue ellipticity and the ellipticity at 222 nm was used to estimate the α-helical content of the peptides [40].

### Alpha-amidation of recombinant wild-type and alanine-substituted Sco-CHH-Gly

The HPLC-purified glycine extended wild-type Sco-CHH (Sco-CHH-Gly), and glycine extended and alanine-substituted Sco-CHHs (I2A, F3A, D4A, D12A, R13A, Q51A, E54A, D60A, I69A, and V72A Sco-CHH-Gly) were C-terminally amidated by peptidyglycine α-amidating mono-oxygenase PAM (Wako) using an established protocol [16]. The amidated products were purified from the reaction mixture using a reversed phase column coupled to the HPLC system and eluted with an elution gradient of ACN containing 10 mM ammonium acetate. Fractions collected were desalted (PD-10, Amersham) and lyophilized [16].

### Mass spectrometric analysis of recombinant wild-type and alanine-substituted Sco-CHH

Mass determination was performed for the amidated recombinant peptides through a commercially available service (Mass Solutions Technology Co., Ltd.) using an LC-MS/MS system with an Accela HPLC station coupled to a Q Exactive Hybrid Quadrupole-Orbitrap Mass Spectrometer (Thermos Scientific). Full mass spectrometry scan was performed with the range of m/z 500–4000 and the isotopically resolved mass spectra further deconvoluted using Thermo Scientific Protein Deconvolution software v2.0.

### Hyperglycemia assay

Experimental animals received a 50-μl injection of crab saline or saline containing recombinant peptides. Procedures for animals handling, sample preparation, and glucose quantification have been described previously [16]. Data were analyzed using Kruskal–Wallis rank sum test with post-hoc Mann-Whitney pairwise comparison (SPSS Manager, SPSS Inc.).

## Results

**Fig 2:**
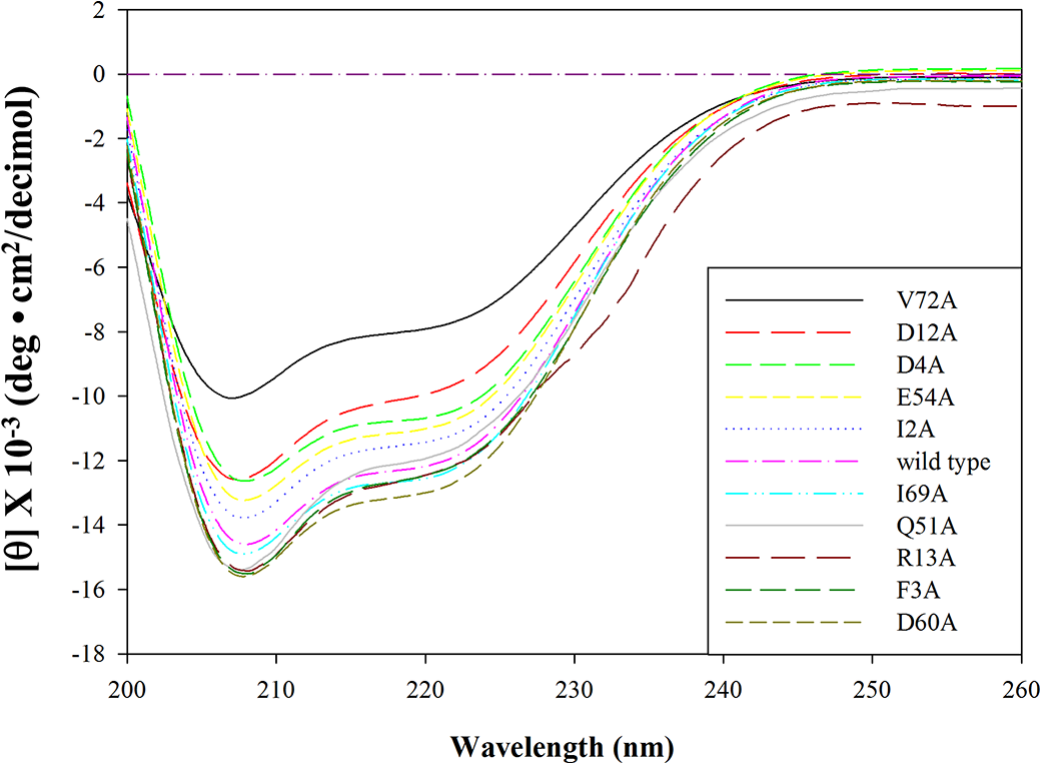
Circular dichroism spectra of wild-type and alanine-substituted rSco-CHH-Gly. CD spectral data of each recombinant peptide, dissolved in 10 mM PBS (150 mM NaCl, pH 6.8), were collected from 260 nm to 200 nm at 25°C using an AVIV 202 spectropolarimeter.

Wild-type, as well as alanine-substituted, Sco-CHHs were produced as glycine-extended recombinant peptides using a bacterial expression system, followed by a refolding reaction and HPLC-purification from the reaction mixture [16]. The refolded and purified peptides (wild-type rSco-CHH-Gly and alanine-substituted rSco-CHH-Gly) were analyzed for their respective circular dichroism (CD) spectra (Fig 2). Profiles of CD spectra of the peptides are similar to one another showing significant negative band at 208 and 222 nm (Fig 2). The estimated proportion of α-helix for Sco-CHH-Gly ranges from 19.7% to 32.4% (Table 1).

**Table 1:** Identification and characterization of wild-type and alanine-substituted rSco-CHH peptides.

rSco-CHH			Mass (Da)^c^	
Peptide	α-helix (%)^a^	elution (% ACN)^b^	Theoretical	Observed
Wild type	30.2	42.6	8522.1	8521.8
I2A	28.5	43.3	8485.8	8484.9
F3A	31.0	43.2	8446.0	8446.8
D4A	32.1	43.8	8483.9	8484.6
D12A	24.6	43.0	8483.9	8482.6
R13A	30.9	43.5	8437.0	8437.8
Q51A	29.6	44.2	8470.8	8471.1
E54A	27.3	42.7	8469.8	8467.7
D60A	32.4	43.9	8483.9	8482.9
I69A	31.4	40.4	8485.8	8486.6
V72A	19.7	41.7	8557.9	8557.0

^a^ Data of % α-helix were calculated from the CD spectral data of peptides not yet amidated.

^b^ Percentage of acetonitrile at which each peptide was eluted.

^c^ Monoisotopic values are given.

All of the recombinant peptides were subsequently subjected to an *in vitro* α-amidation reaction. The resulting amidated peptides (wild-type rSco-CHH and alanine-substituted rSco-CHH) were HPLC-purified from the reaction mixtures. The percentage of acetonitrile (% ACN) where wild-type Sco-CHH was eluted is 42.6% and alanine-substituted Sco-CHHs were eluted within the range of 40.4%–44.2% ACN (Table 1). Mass spectrometric analyses of the recombinant peptides resulted in experimentally observed mass values that agree with the corresponding theoretical values (Table 1).

**Fig 3:**
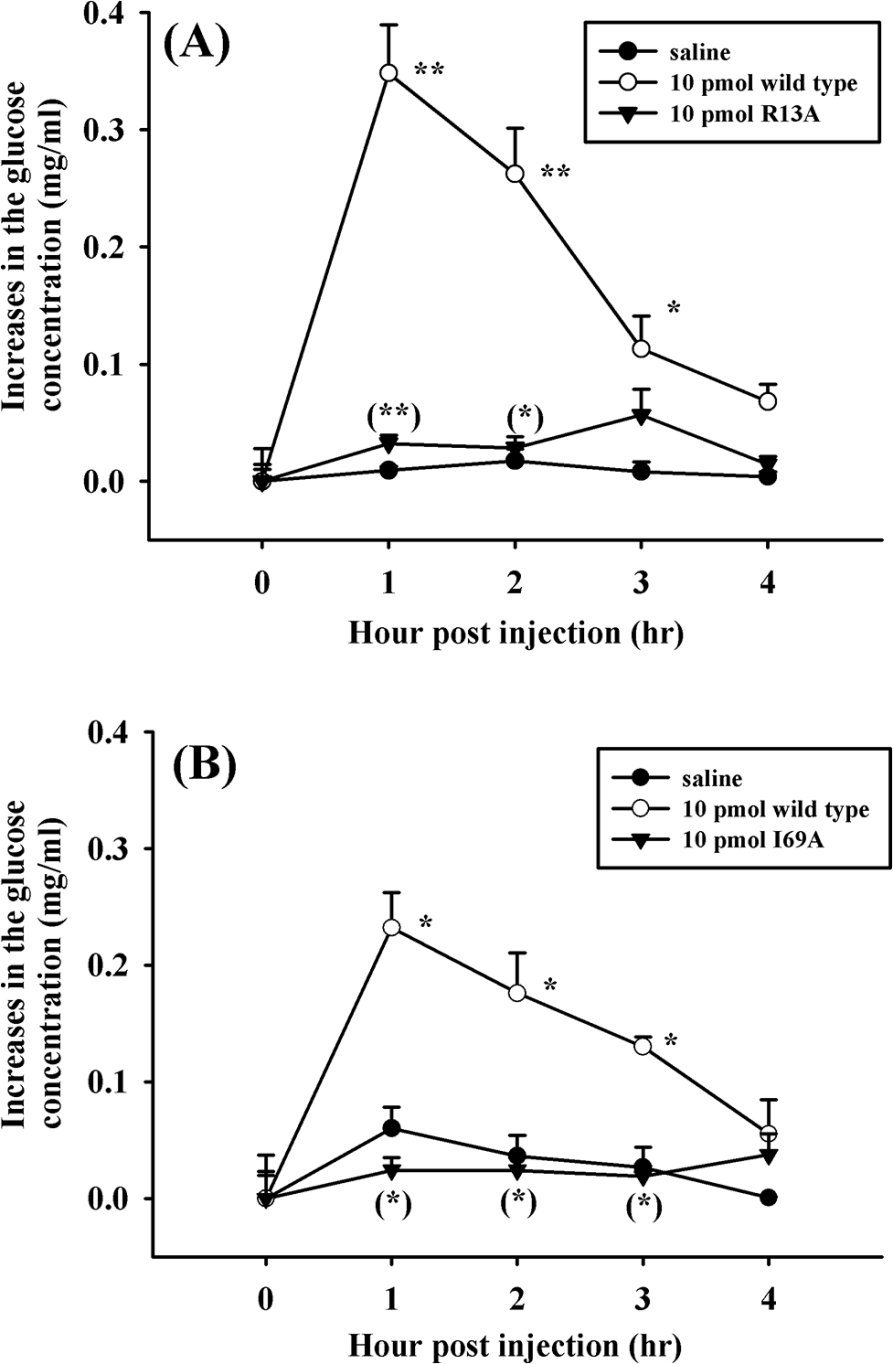
Two recombinant Sco-CHH mutants completely lose hyperglycemic activity. Eyestalk-ablated animals (*S*. *olivacea*) received a 50-μl injection of (A) R13A rSco-CHH (▼: 10 pmol/animal), (B) I69A rSco-CHH (▼: 10 pmol/animal), (A, B) wild-type rSco-CHH (○: 10 pmol/animal) or saline (●). Hemolymph was withdrawn at designated time points and processed for determination of glucose levels. Data are given as mean ± S.E.M. For the sake of clarity, one-sided SEM bars are given. Sample size (n) is 8 for each time point. *,** indicate significant differences from corresponding zero time controls at 5% and 1% levels, respectively. Parenthesized *,** indicate significant differences between the wild-type and mutants at the same time-point at 5% and 1% levels, respectively. No significant change over time was observed in saline-treated animals.

Alanine-substituted Sco-CHHs were then tested for hyperglycemic activity concurrently against wild-type Sco-CHH (a positive control) and the vehicle treatment (a negative control). Two aniline-substituted and amidated mutants (R13A and I69A rSco-CHH) completely lacked hyperglycemic activity. Thus, while wild-type Sco-CHH significantly increased hemolymph glucose levels at 1 h post injection (hpi) and the glucose levels remained significantly higher than the pre-injection (0 h) levels at both 2 and 3 hpi, neither R13A nor I69A Sco-CHH at any time point significantly changed the glucose levels, with the temporal profiles similar to that of the vehicle treatment (Fig 3).

**Fig 4:**
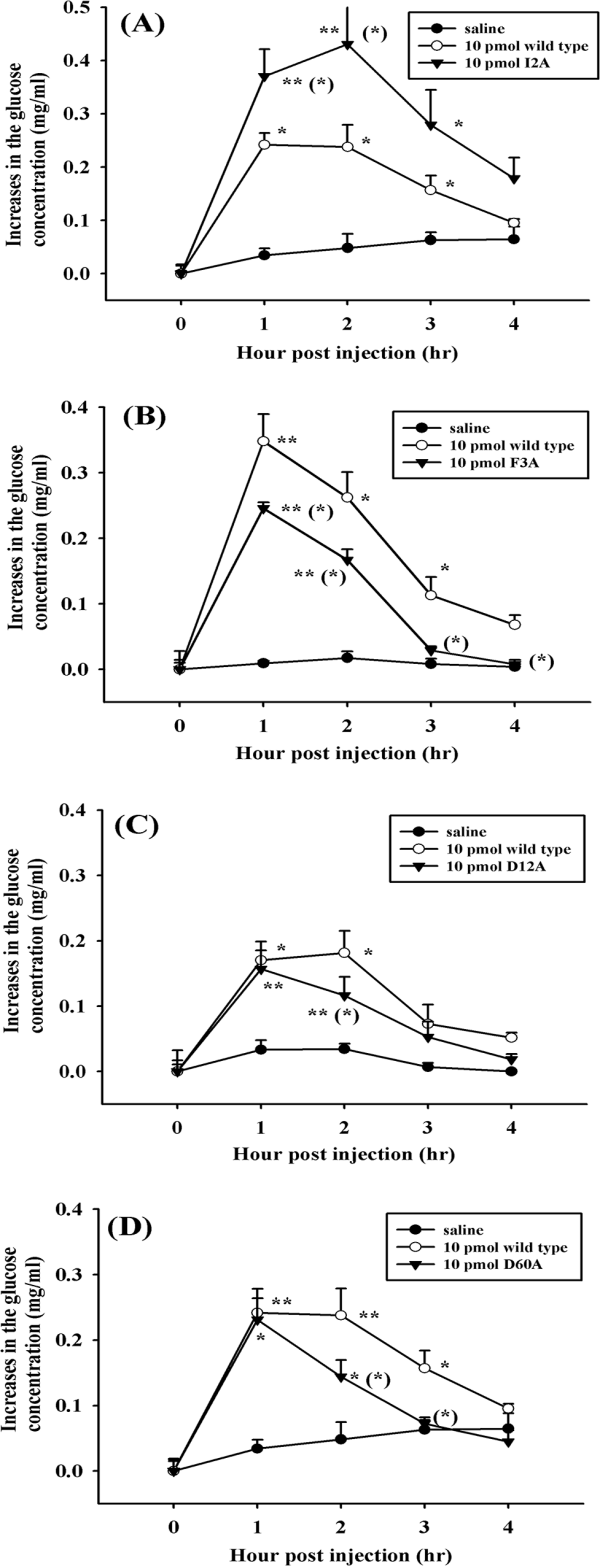
Four recombinant Sco-CHH mutants exhibit hyperglycemic activities significantly different from those elicited by wild-type Sco-CHH. Eyestalk-ablated animals (*S*. *olivacea*) received a 50-μl injection of (A) I2A rSco-CHH (▼: 10 pmol/animal), (B) F3A rSco-CHH (▼: 10 pmol/animal), (C) D12A rSco-CHH (▼: 10 pmol/animal), (D) D60A rSco-CHH (▼: 10 pmol/animal), (A-D) wild-type rSco-CHH (○: 10 pmol/animal) or saline (●). Hemolymph was withdrawn at designated time points and processed for determination of glucose levels. Data are given as mean ± S.E.M. For the sake of clarity, one-sided SEM bars are given. Sample size (n) is 8 for each time point. *,** indicate significant differences from corresponding zero time controls at 5% and 1% levels, respectively. Parenthesized * indicates significant differences between the wild-type and mutants at the same time-point at 5%. No significant change over time was observed in saline-treated animals.

The temporal profiles of hyperglycemic responses elicited by 4 aniline-substituted mutants, *i*.*e*., I2A, F3A, D12A, and D60A Sco-CHH, were different from those elicited by wild-type Sco-CHH. Among these mutants, I2A Sco-CHH is the only one that increased the hemolymph glucose levels, at 1 and 2 hpi, that were significantly higher than those increased by wild-type Sco-CHH (Fig 4). On the other hand, although F3A, D12A, and D60A Sco-CHH, similar to wild type Sco-CHH, significantly elevated glucose levels at 1 and 2 hpi when compared to their corresponding pre-injection levels (with the hyperglycemic responses elicited by D12A and D60A Sco-CHHs at 1 hpi being comparable to the levels by wild-type Sco-CHH), the glucose levels elevated by these mutants declined faster than those by wild-type Sco-CHH (Fig 4). Thus, the glucose levels increased by the mutants were significantly lower than the wild-type levels at 2 hpi (D12A), at 2 and 3 hpi (D60A), or at 1, 2, 3, and 4 hpi (F3A) (Fig 4).

**Fig 5:**
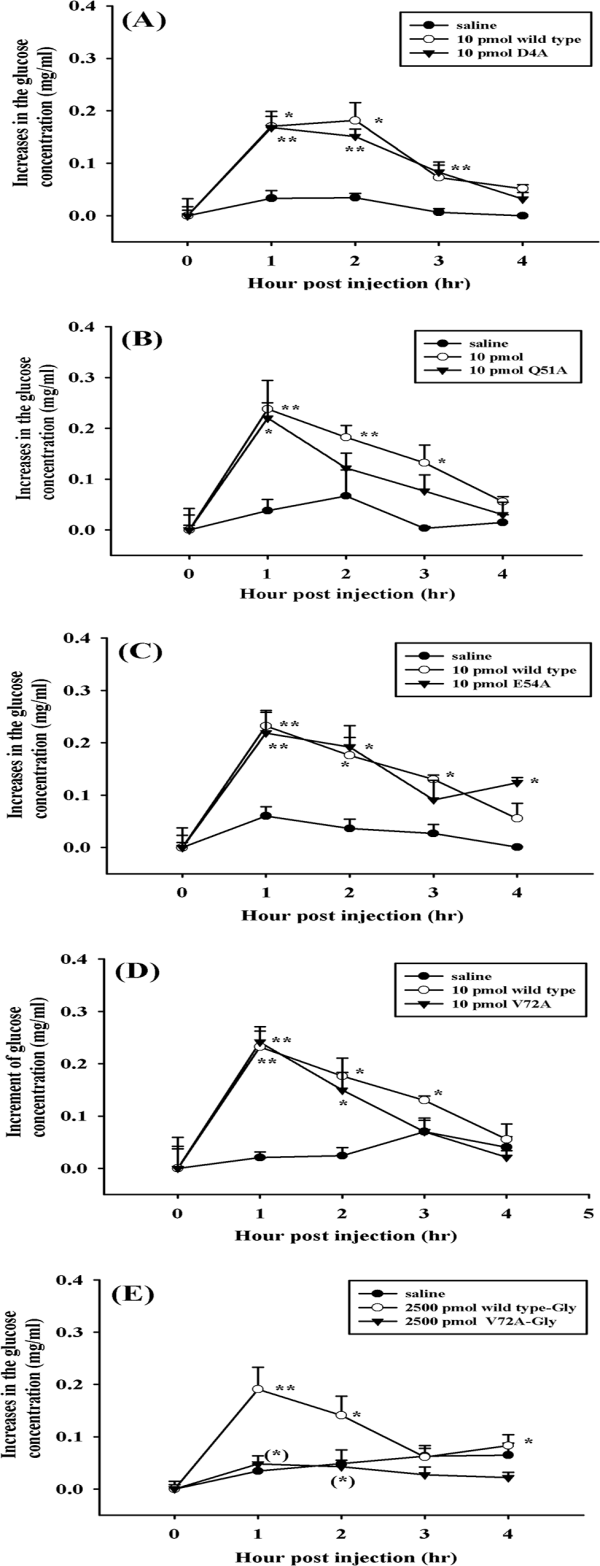
Four recombinant Sco-CHH mutants exhibit hyperglycemic activities not significantly different from those elicited by wild-type Sco-CHH. Eyestalk-ablated animals (*S*. *olivacea*) received a 50-μl injection of (A) D4A rSco-CHH (▼: 10 pmol/animal), (B) Q51A rSco-CHH (▼: 10 pmol/animal), (C) E54A rSco-CHH (▼: 10 pmol/animal), (D) V72A rSco-CHH (▼: 10 pmol/animal), (E) V72A rSco-CHH-Gly (▼: 2500 pmol/animal), (A-D) wild-type rSco-CHH (○: 10 pmol/animal), (E) wild-type rSco-CHH-Gly (○: 2500 pmol/animal) or (A-E) saline (●). Hemolymph was withdrawn at designated time points and processed for determination of glucose levels. Data are given as mean ± S.E.M. For the sake of clarity, one-sided SEM bars are given. Sample size (n) is 8 for each time point. *,** indicate significant differences from corresponding zero time controls at 5% and 1% levels, respectively. Parenthesized * indicates significant differences between the wild-type and mutants at the same time-point at 5%. No significant change over time was observed in saline-treated animals.

Four alanine-substituted mutants, D4A, Q51A, E54A, and V72A rSco-CHH, elicited hyperglycemic responses with temporal profiles similar to those evoked by wild-type Sco-CHH, without statistically significant difference at any time points (Fig 5A–5D). In contrast, the glycine-extended version of V72A rSco-CHH, V72A rSco-CHH-Gly, even at a very high dose (2500 pmol/animal), lacked hyperglycemic activity, with the temporal profile similar to that of the vehicle treatment (Fig 5E).

## Discussion

### Selection of mutation sites

Ten sites of *Scylla olivacea* CHH (Sco-CHH) [41] were chosen for site-directed mutagenesis (Fig 1). The chosen sites for mutation are located in the N- and C-termini of Sco-CHH, mainly in motifs A1, A4 and A5 of Group I (which includes CHH and ITP), and are considered to be functionally relevant [5,30]–Phe^3^ (F3), Asp^4^ (D4), Asp^12^ (D12), Arg^13^ (R13) are located in motif A1, Gln^51^ (Q51) and Glu^54^ (E54) in motif A4; Ile^69^ (I69) in motif A5; Val^72^ (V72) is a well-conserved C-terminal residue, amidation of which has been shown to be essential for hyperglycemic activity of CHH [16,27,29,42], Ile^2^ (I2) aligns with Phe^2^ of Scg-ITP, which has been shown to be important for ITP activity [35], and Asp^60^ (D60) is located at a position, where there is predominantly a negatively charged residue in the peptides of Group I.

### Preparation and characterization of recombinant peptides

Ten alanine-substituted mutants of CHH peptides, as well as wild-type peptide, were expressed using an *E*. *coli* system, refolded, and purified. The resulting glycine-extended recombinant peptides (wild-type and mutated rSco-CHH-Gly) were subjected to a circular dichroism (CD) spectral analysis, followed by a reaction of C-terminal amidation; the amidated peptides were then determined for their masses by a mass spectrometric analysis.

The CD spectra of wild-type and mutated rSco-CHH-Gly, with significant negative bands at 208 and 222 nm (Fig 2), are similar to that of native Sco-CHH [16]. Calculated proportions of α-helix for the recombinant peptides are similar to one and other (24.6%–32.4%), except V72A rSco-CHH-Gly (19.7%) (Table 1); but they are lower than that of native Sco-CHH (41%) [16]. Deviation of the glycine-extended recombinant peptides from native peptide in α-helical content is likely due to the fact that the formers have not yet been amidated. A previous study of ours showed that, after α-amidation, wild-type rSco-CHH increased its helical content to a level (47%) comparable to that of native Sco-CHH [16]. Percentage of acetonitrile (% ACN) at which the amidated rSco-CHHs were eluted during a gradient elution of chromatographic separation were close to one and other (40.4%–44.2%, Table 1), as well as to that of native Sco-CHH (42%) [16]. The combined results indicate that the recombinant peptides were properly expressed and refolded, to the extent that they are similar to native Sco-CHH in conformation. Finally, the observed value of mass of each rSco-CHH closely agrees with the theoretical value calculated from the peptide sequence (Table 1), suggesting that mutants with the intended substitutions were produced. The recombinant peptides were then assessed for hyperglycemic activity using an established *in vivo* assay [16].

### Functional analyses of N-terminal residues

**Fig 6:**
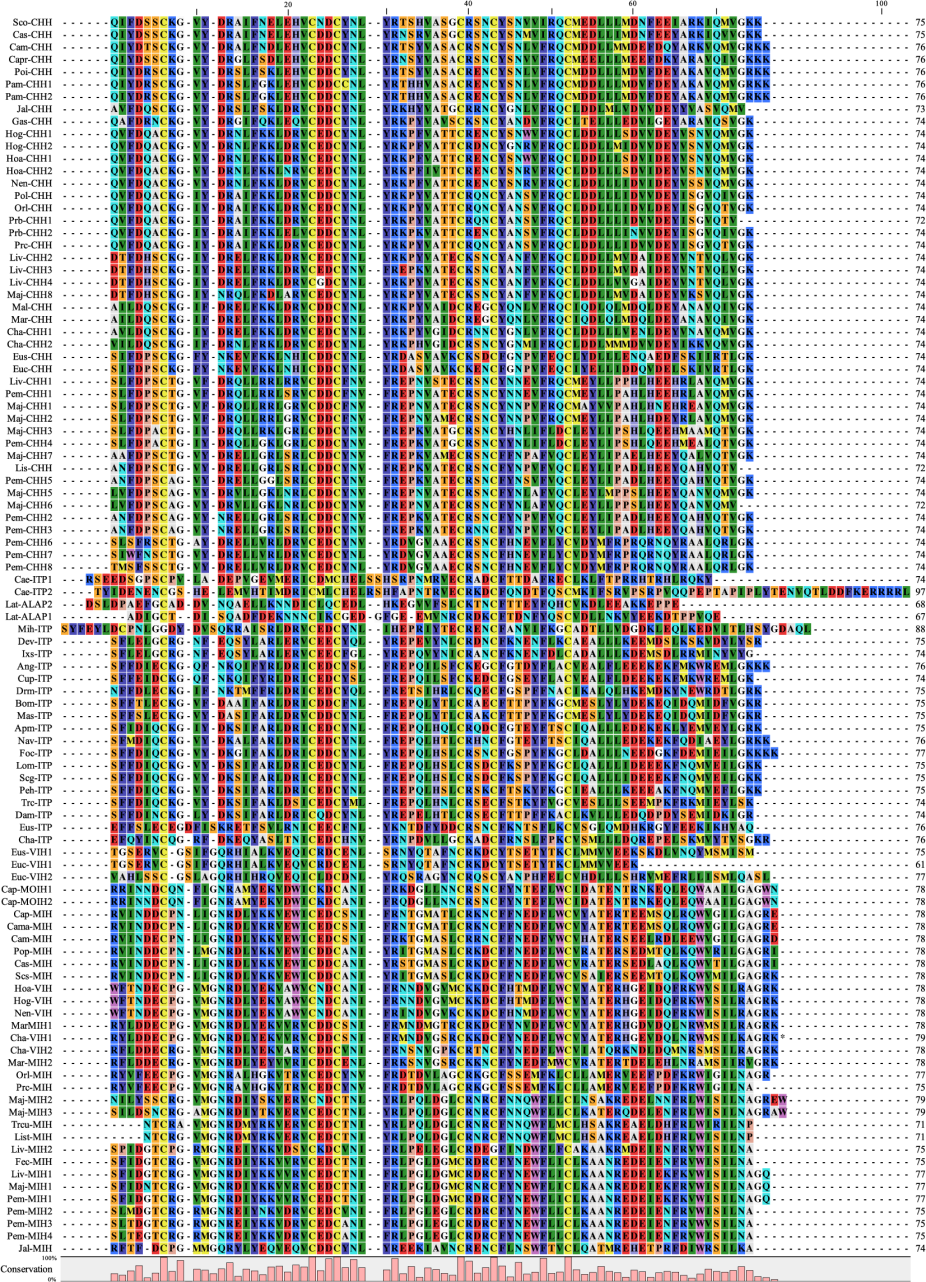
Multiple sequence alignment of representative peptides of the CHH family. Residues are numbered according to Sco-CHH. Sequences aligned are from the following sources: *Anopheles gambiae*: Ang-ITP (sequence deduced from genomic sequences, 9); *Apis mellifera*: Apm-ITP (XP_001120062); *Bombyx mori*: Bom-ITP (AAY29659); *Caenorhabditis elegans*: Cae-ITP1 (Q23247), Cae-ITP2 (NP_508782); *Cancer magister*: Cama-MIH (O61389); *Callinectes sapidus*: Cas-CHH (AAS45136), Cas-MIH (P55321); *Carcinus maenas*: Cam-CHH (P14944), Cam-MIH (Q27225); *C*. *productus*: Capr-CHH (ABQ41269), Cap-MOIH1 (CAC05347), Cap-MOIH2 (CAC05348), Cap-MIH (CAC05346); *Chorismus antarcticus*: Cha-CHH1, Cha-CHH2, Cha-ITP, Cha-VIH1, Cha-VIH2 (unpublished data); *Culex pipens*: Cup-ITP (XP_001845654); *Daphnia magna*: Dam-ITP (ABO43964); *Dermacentor variabilis*: Dev-ITP (ACC99599); *Drosphila melanogaster*: Drm-ITP (ABZ88142); *Euphausia crystallorophias*: Euc-CHH, Euc-VIH1, Euc-VIH2 (*E*. *crystallorophias* ERP002510); *E*. *superba*: Eus-CHH, Eus-ITP; Eus-VIH1 (*E*. *superba* transcriptome SRA023520); *Fenneropenaeus chinensis*: Fec-MIH (AAL55258); *Folsomia candida*: Foc-ITP (ACF15252); *Galathea strigose*: Gas-CHH (ABS01332); *Homarus gammarus*: Hog-CHH1 (ABA42179), Hog-CHH2 (ABA42180), Hog-VIH (ABA42181); *H*. *americanus*: Hoa-CHH1 (P19806), Hoa-CHH2 (Q25154), Hoa-VIH (P55320); *Ixodus scapularis*: Ixs-ITP (sequence deduced from genomic sequences, 33); *Jasus lalandii*: Jal-CHH (P56687), Jal-MIH (P83220); *Latrodectus tredecimguttatus*: Lat-ALAP2 (Q4U4N3), Lat-ALAP1 (P49125); *Litopenaeus vannamei*: Liv-CHH1 (Q26181), *L*. *stylirostris*: Lis-CHH (P59685), Lis-MIH (AAL55257); Liv-CHH2 (AAN86055), Liv-CHH3 (S73824), Liv-CHH4 (EF156402), Liv-MIH1 (AAR04348), Liv-MIH2 (AAR04349); *Locusta migratoria*: Lom-ITP (AAD20820); *Marsupenaeus japonicus*: Maj-CHH1 (O15908), Maj-CHH2 (Q9U5D2), Maj-CHH3 (Q94676), Maj-CHH5 (O15981), Maj-CHH6 (P81700), Maj-CHH7 (O15982), Maj-CHH8 (AB247560), Maj-MIH1 (P55847), Maj-MIH2 (BAD36757), Maj-MIH3 (BAE78494); *Macrobrachium lanchesteri*: Mal-CHH (O77220); *M*. *rosenbergii*: Mar-CHH (AF219382), Mar-MIHH1 (AAL37948), Mar-MIH2 (AAL37949); *Manduca sexta*: Mas-ITP (AAY29657); *Microctonus hyperodae*: Mih-ITP (ABY19395); *Nasonia vitripenis*: Nav-ITP (XP_001604056); *Nephropas norvegicus*: Nen-CHH (AAQ22391), Nen-VIH (AAK58133); *Orconectes limosus*: Orl-CHH (Q25589), Orl-MIH (P83636); *Pachygrapsus marmoratus*: Pam-CHH1 (AAO27804), Pam-CHH2 (AAO27805); *Pediculus humanus corporis*: Peh-ITP (AEEB14555); *Penaeus monodon*: Pem-CHH1 (O97383), Pem-CHH2 (Q97384),Pem-CHH3 (Q97385), Pem-CHH4 (O97386), Pem-CHH5 (O97387), Pem-CHH6 (AAQ24525), Pem-CHH7 (AAQ24526), Pem-CHH8 (AAQ24527), Pem-MIH1 (AAR89516), Pem-MIH2 (AAR89517), Pem-MIH3 (BAB69829), Pem-MIH4 (BAB69830); *Pontastacus leptodactylus*: Pol-CHH (AAX09331); *Portunus pleagicus*: Pop-MIH (ABM74397); *Potamon ibericum*: Poi-CHH (ABA70560); *Procambarus bouvieri*: Prb-CHH1 (P55845), Prb-CHH2 (Q10987); *P*. *clarkii*: Prc-CHH (Q25683), Prc-MIH (P-55848); *Schistocerca gregaria*: Scg-ITP (AAB16822); *Scylla olivacea*: Sco-CHH (AAQ75760); *Scylla serrta*: Scs-MIH (AAL99355); *Trachypenaeus curvirostris*: Trcu-MIH (AAL55259); *Tribolium castaneum*: Trc-ITP (ABN79658).

In N-terminus, it has been found that 4 mutants (I2A, F3A, D12A, and R13A) significantly altered its hyperglycemic activity and 1 mutant (D4A) did not (Figs 3, 4 and 5). Among the alanine substitutions that led to changes, in terms of potency and time course, in hyperglycemic activity, I2A rSco-CHH is unique in that it elicited a hyperglycemic response significantly higher than that by wild-type rSco-CHH. For an insect ion-transport peptide, Scg-ITP, it was found that Phe^2^ is essential for a biological activity of ITP, *i*.*e*., evoking an active Cl^-^ transport across the ileum; alanine substitution for Phe^2^ in Scg-ITP resulted in complete loss of activity [35], in contrast to the observation that alanine substitution for Ile^2^ in Sco-CHH significantly increased hyperglycemic activity. These results indicate that the second residue of CHH and ITP interacts differently with their receptors, possibly because the 2^nd^ residues in CHH and ITP are of different characteristic. Interestingly, the majority of CHH reported thus far, like Sco-CHH, has at the second position a hydrophobic residue without aromatic ring (Ile, Leu, Val, or Ala), whereas ITP always has a Phe^2^ (Fig 6), which has an aromatic side chain. This suggests that a residue with aromatic side chain at 2^nd^ position is essential for ITP activity but not required for CHH activity. Thus, experimental evidence supports the suggestion that characteristic of the second residue (*i*.*e*., hydrophobic residue with or without aromatic side chain) is a factor responsible at least in part for functionally differentiating CHH from ITP. Exceptions to the conserved property at this position occur mainly in penaeid CHHs–Lis-CHH in *Litopenaeus stylirostris*, Pem-CHH2, 3, 5 in *Penaeus monodon*, Liv-CHH2, 3, 4 in *L*. *vannamei*, and Maj-8 in *Marsupenaeus japonicas* have a polar (but uncharged) residue (Asn or Thr). Nonetheless, other CHHs (Pem-CHH1, 4, Liv-CHH1) discovered in 2 of these species also have a hydrophobic residue without aromatic side chain, like the majority of CHH. These exceptions and several ones at other positions (see Fig 6) will be discussed later.

A D-form, instead of an L-form, of Phe^3^ is present in the so-called D-Phe^3^ CHH isolated from the sinus glands of several astacideans [23,43,44,45,46,47]. D-Phe^3^ CHH, when compared to all-L CHH, has been shown to be more potent in hyperglycemic activity, molt-inhibiting activity, or both [23,48,49]; presumably the two CHH isoforms have different half-life in circulation, different receptor-binding characteristics, or both [46,48,49]. Further, although D-Phe^3^ CHH has not been reported for any crustacean other than astacideans, Phe^3^ appears in the majority of CHH (Fig 6). Together, these data imply that Phe^3^ is a functionally important residue for CHH. Indeed, the suggestion is supported by the observation that F3A rSco-CHH exhibited significantly lower hyperglycemic activity than wild-type Sco-CHH, at all the time points sampled after injection. Similarly, a recent study also demonstrated that D-alanine substitution for Phe^3^ of CHH (Glp-D-A-CHH) in the crayfish *Pontastacus leptodactylus*, reduced its hyperglycemic activity, when compared to the wild-type counterpart, with either a D-Phe^3^ or an L-Phe^3^ (*i*.*e*., Glp-D-CHH or Glp-L-CHH) [50]. Both ITP and CHH are quite conserved at position 3, almost always having Phe^3^ (ITP), Phe^3^ or Tyr^3^ (CHH) (Fig 6), demonstrating that for both peptides a residue with aromatic side chain is required for activity.

D4A substitution in Sco-CHH did not result in change of activity, agreeing with the result of D4A substitution in Scg-ITP [35]. These results are unexpected as, for both CHH and ITP, predominantly a negatively charged residue (Asp^4^ or Glu^4^) appears at position 4 (Fig 6). Nonetheless, it was suggested that the conserved Asp^4^ is required for a yet uncharacterized function of ITP [35]. It is widely considered that CHH is pleiotropic, possibly having functions other than eliciting hyperglycemic responses (see [31,32] for reviews); thus, it is entirely plausible that Asp^4^ in CHH might be essential for function(s) other than hyperglycemic activity.

In Group-I peptides, there is predominantly an Asp at position 12, and alanine substitution for Asp^12^ in Sco-CHH significantly decreased hyperglycemic activity. Similarly, a D12N mutant tested in a study of the Norway lobster *Nephrops norvegicus* [37] was found to have significantly reduced its hyperglycemic activity. R13A rSco-CHH completely lost its hyperglycemic activity. Its importance for the hyperglycemic activity of CHH is well-expected since Arg^13^ (or another positively charged residue, Lys^13^) is conserved family-wide, appearing in both Group-I and-II peptides (Fig 6). No corresponding mutant has been tested for Scg-ITP (Table 2), which also has Asp^12^ and Arg^13^ (Fig 6).

**Table 2:** Position and residue of CHH, ITP, MIH peptides which point-mutated peptides have been tested for importance in biological activity.^a^

	Motif and residue number^b^										
		A1/A1'				A4/A4'			A5		
	2	3	4	12	13	51	54	55	60	69	72
				(13)	(14)	(52)		(56)			(73)
Peptide											
Sco-CHH	I2A ^c^	F3A	D4A	D12A	R13A	Q51A	E54A	D	D60A	I69A	V72A-NH2^d^
											V72A ^e^
Scg-ITP	F2A	F3A	D4A	D	R			A		V	L72A_-_NH2^d^
											L72A ^e^
Maj-MIH				N13A	R14A			A56Y		I	L

^a^ Data are summarized from the present study (Sco-CHH), [34,35] (Scg-ITP), and [33] (Maj-MIH). Note that for ITP and MIH, only mutants and residues relevant for discussion are presented.

^b^ Non-parenthesized number indicates the position of the residue for CHH and ITP sequences, and parenthesized number that for MIH sequence. Underlined number indicates position of the residues that is suggested to be functionally discriminating Group I and Group II peptides, with the exceptions that residue at position 2 being suggested to be functionally discriminating CHH and ITP and that residues at positions 55/56 among CHH, ITP, and Group II peptides. Motif names in which the residues are located according to Lacombe et al. [30] are given above residue numbers.

^c^ Underlined mutant indicates significant change in activity, with all mutants exhibited no activity or activity lower than wild-type, except I2A, which exhibited higher activity; single letter indicates the residue at that position.

^d,e^ amidated and non-amidated mutants, respectively.

When compared to Group-II peptides (VIH, MIH, and MOIH) [30], it was noted that Asp^12^ (a negatively charged residue) in Group-I peptides is replaced at the aligned position by Asn^13^ or Gln^13^ (polar but uncharged residues) in Group II; Gly^12^ in Group II is missing in Group I (Fig 6; see also [30]). For Asp^12^ in Sco-CHH and Asn^13^ in Maj-MIH, the importance of the residues in affecting respective activity of CHH and MIH was confirmed by testing the effect of alanine- or asparagine-substituted mutants (Table 2, the present study; [33]). Conversely, Δ12, a mutated Maj-MIH, with Gly^12^ being deleted, did not significantly change its molt-inhibiting activity, when compared to wild-type MIH [33]. In addition, property-conserved residues (Phe^3^ or Tyr^3^) at position 3 of Group-I peptides, which as mentioned above have been experimentally confirmed to be functionally relevant for Sco-CHH and Scg-ITP (the present study; 58), are absent from the N-terminus of Group-II peptides, where motif A1' (a Group II-specific motif) is located [30]. In Group-II peptides, residue with aromatic side chain never occurs at position 3.

The combined experimental data derived from studies of Sco-CHH, Scg-ITP, and Maj-MIH and those from sequence alignment ([27,35]; the present study) regarding N-terminus identified several group-specific and functionally critical residues in and around motifs A1 and A1′ (see Table 2)–Phe^3^ (and most likely also Asp^12^) are shared residues critical for the activity of Group-I peptides (CHH and ITP); side chain property of the second residue (Ile^2^/Phe^2^) is an important feature in functionally differentiating CHH from ITP. Moreover, presence (or absence) of Phe^3^ and changes in residue property (Asp^12^/Asn^13^) contribute to functional differentiation between peptides of Groups I and II (Table 2).

### Functional analyses of C-terminal residues

A CHH structural variant, CHH-like peptide (CHH-L), has been purified from the pericardial organs of 3 brachyurans and the thoracic ganglia of an astacidean [16,19,47,51]. CHH and CHH-L are products of alternatively spliced transcripts [19,41,51,52,53]; the 2 CHH variants share an identical N-terminal sequence (residues up to the 40^th^), but differ considerably in the remaining sequence and are functionally different in that CHH-L neither exhibits hyperglycemic activity nor suppresses secretion of ecdysteroids by the molting gland [16,19,42]. Obviously, residues in the remaining C-terminus of CHH are also required for full exhibition of hyperglycemic activity.

In C-terminus, residues of Sco-CHH located mainly in motifs 4 and 5 of Group-I peptides were tested. Q51A and E54A substitutions did not significantly alter hyperglycemic activity of Sco-CHH, which are rather unexpected as Gln^51^ is quite well-conserved for CHH and at position 54 there is almost always a negatively charged residue, Asp^54^ or Glu^54^ (Fig 6). Like Asp^4^ discussed above, both residues might be essential for function(s) other than hyperglycemic activity. In addition, residue at the position of 51 is one of the residues in motif A4 that possibly functionally discriminates CHH from ITP, with the predominantly polar residue (Gln^51^) in CHH being changed to residues of variable property (mainly hydrophobic or non-polar residue) in ITP (see Fig 6). Similarly, as stated above, residue at position of 54 is almost always a negatively charged residue in CHH; but it is more variable in ITP (mainly polar or negatively charged residue). However, neither Q51A nor E54A rSco-CHH changed hyperglycemic activity and no corresponding ITP mutant has been experimentally tested (Table 2).

D60A rSco-CHH evoked hyperglycemic responses at least 2 times lower than did wild-type rSco-CHH. The majority of CHHs has an Asp^60^ or Glu^60^, both negatively charged residues (Fig 6). For ITP, residue at this position is also usually negatively charged (Asp^60^ or Glu^60^), but no corresponding ITP mutant has been tested for its activity.

As stated above, position 69 is located in motif 5 of Group-I peptides. The observation that I69A Sco-CHH completely lacked hyperglycemic activity is interesting. Sco-CHH is among only a few CHHs where Ile is present at this position (the others being Cas-EG-CHH from *C*. *sapidus*), instead of Val for almost all other CHHs (see Fig 6). It appears that a hydrophobic amino acid at this position is important for hyperglycemic activity of CHH. However, a substitution for Ile69 of Ala, also a hydrophobic residue but with a smaller side chain, resulted in loss of activity strongly argues that the size of the hydrophobic side chain at position 69 is critical for the activity of CHH. No corresponding ITP mutant has been tested for its activity, although a hydrophobic residue (Val^69^ or Ile^69^) is also present in Scg-ITP and other ITPs at this position (Fig 6; see also [30]).

It was found that V72A rSco-CHH, but not V72A rSco-Gly (Fig 5D and 5E), exhibited hyperglycemic activity, which reiterates the importance of the amide moiety at the C-terminal end for the hyperglycemic activity of CHH, a conclusion reached by studies of several species where wild-type rCHH-Gly and wild-type rCHH were tested and compared [16,27,29]. However, functional importance of the side chain of Val^72^ has not been fully tested. Valine^72^ is very well-conserved in CHH, and V72A rSco-CHH is a mutant with alanine substitution for valine, both are hydrophobic residues with a relatively small side chain. Nonetheless, it is observed that V72A rSco-CHH-Gly has a much lower α-helical percentage than its wild-type counterpart; in fact, it is also lower than all other alanine-substituted mutants (Table 1); we also observed that, although a very high dose (2500 pmol/animal) of wild-type rSco-CHH-Gly was able to evoke a significant hyperglycemic activity, V72A rSco-CHH-Gly at the same dose was not (Fig 5E). It appears that the side chain of Val^72^ might also be important for hyperglycemic activity of CHH. To fully assess the importance of the side chain of Val^72^, a mutant substituted by a residue with a charged or a larger hydrophobic side chain could be tested.

Both studies of CHH [16,27,29,54] and ITP [34] showed that an amidated 72^nd^ residue is required for full activity. The present study and that of Wang et al. [34] further showed that alanine substitution for the 72^nd^ residue (V72A rSco-CHH and KcITP_A72_, respectively) did not significantly alter their respective activity. These results again emphasize that the C-terminal amide moiety is essential for the function of both CHH and ITP.

Motif A4 in Group I differs from motif A4' Group II at the 3^rd^ and 7^th^ positions of the motifs, with respect to side-chain property (see [30]). They correspond to the 51^st^ and 55^th^ residues (Gln^51^ and Asp^55^, which are polar and negatively charged, respectively) in rSco-CHH and 52^nd^ and 56^th^ residues (Ile^52^ and Ala^56^, both are hydrophobic) in Maj-MIH (Fig 6 and Table 2). The predominantly polar residue (Gln^51^) in CHH is changed to residues of variable property (mainly hydrophobic or non-polar residue) in Group-II peptides (Fig 6). However, Q51A rSco-CHH did not change its activity, when compared to wild-type rSco-CHH (the present study), and corresponding MIH mutant has not been tested. On the contrary, A56Y MIH reduced its activity by an order of one magnitude [33]; thus, at least the result of A56Y MIH is not incompatible with the suggestion that a change between negatively charged and hydrophobic residues occurring at 55^th^/56^th^ residue might contribute to divergence of CHH and MIH activity. At position 55, CHH usually has a negatively charged residue (Asp^55^ or Glu^55^) and ITP a hydrophobic or polar residue (Ala^55^, Val^55^, Ser^55^), and at position 56 (Ala^56^) Group-II peptide mainly has a hydrophobic residue (Ala^56^). Further, it is known that, in contrast to Group-I peptides, Group-II peptides are free in the C-terminal end without amidation; in addition, an MIH mutant (Δ75–77 with the last 3 residues being deleted) did not exhibit changed activity [33].

Several conclusion can be made based on the combined data of CHH, ITP, and MIH studies ([33,34] and the present study) regarding the C-terminus (see Table 2). It is clear that the C-terminal amide moiety and likely a local environment of hydrophobicity provides by certain residues (*e*.*g*., position 69, 72, or both) is critical for the function of Group-I peptides; negatively charged residue at position 60 is likely also functionally required. For functionally discriminating residues, 55^th^/56^th^ residue is likely one that distinguishes among CHH, ITP, and Group-II types, and the presence or absence of an amidated end respectively in Group I and Group II is another responsible for the diversification of function between peptides of the 2 groups.

Finally, but not least importantly, it is noted that residues at the position of conserved property discussed above exhibit property variability, occurring mainly in penaeid and palaemonid CHH sequences and diptera ITP sequences. For example, at position 60, for CHH the predominantly negatively charged residue (Asp^60^ or Glu^60^) is replaced by a polar residue in Pem-CHH4, Maj-CHH5, 6, Eus-CHH, a hydrophobic residue in Liv-CHH4, or a positively charged residue in Liv-CHH1, Pem-CHH1, 6, 7, 8, Maj-CHH1, 2, 3. However, conforming residues are also present in certain CHH isoforms (*e*.*g*., Liv-CHH2, 3, Pem-CHH2, 3, 5, Maj-CHH7, 8) of these species, with a negatively charged residue at position 60 (Fig 6). Interestingly, for CHHs in *M*. *japonicus*, the 2 with a polar residue (Ser^60^ in Maj-CHH5, 6) appeared exhibiting higher maximal hyperglycemic activity than those with a positively charged residue (His^60^ in Maj-CHH1, 2, 3) [55]. These results suggest that, at least in penaeids where many CHH isoforms have been frequently observed (*e*.*g*., up to 8 CHHs in *P*. *monodon* and to 7 CHHs in *M*. *japonicas* have been recorded), variation in residue property at the “conserved positions” among different isoforms, which possibly arose through gene duplication and mutation, might lead to different levels of hyperglycemic activity (or perhaps even different function) (see [56] for review), a suggestion that again highlights the functional importance of the residue at these positions. Another 2 examples concern insect ITP. Like CHH, most ITP have a negatively charged residue (Asp^12^) at position 12, but exceptions occur in at least 3 diptera ITPs (Ang-ITP, Cup-ITP, Drm-ITP) that have polar residue (Asn^12^) at this position; likewise, the residues at position 69 of CHH and ITP are usually hydrophobic, but the same 3 diptera ITPs have positively charged residue (Arg^69^). The significance of certain ITPs “deviate” from the others in terms of conservation of residue property is not known. An earlier study had demonstrated that the extracts of head (including brains and corpora cardiaca) in insect species (including dipterans) not phylogenetically closely related to locusts were inactive in the locust ileal bioassay, suggesting sequence divergence between insect orders [57].

## Conclusion

In summary, the present study, the first comprehensive study of CHH screening functionally important residues, have provided experimental evidence demonstrating several critical residues in both termini of Sco-CHH. More importantly, comparing our study with previous ones of ITP and MIH, first of all, reaches a conclusion that CHH and ITP, both members of Group I, share several residues of conserved property in or around motifs A1 and A5 with functional importance. It should be noted however that a recent phylogenetic study created a third group (Group III) of the CHH family for all insect ITPs and other related non-arthropod peptides [9], indicating that sequence characteristics exist that justify separation of ITP from CHH. Furthermore, CHH did not exhibit activity in the insect ileal assay [58] and that both CHH and ITP are recently found to be present in the same species (*i*.*e*., Cha-CHH1, Cha-CHH2, Cha-ITP of the Antarctic shrimp *Chorismus antarcticus* and Eus-CHH, Eus-ITP of the Antarctic krill *Euphausia superba*) for the first time (see Fig 6). Assuming CHH and ITP each exerts its distinct function through interacting with different receptor, there should be residues responsible for functional divergence between the 2 peptides of Group I. In this regard, the second position is the only one where residues (Ile^2^/Phe^2^) have been experimentally demonstrated to be implicated in functionally differentiating CHH from ITP. Thirdly, residues discriminating Group-I from Group-II peptides include characteristic residues in or around motifs A1/A1′, A4/A4′, A5/A5′. With novel functions being ascribed to CHH-family peptides, and with more CHH-family peptides with peculiar sequence characteristic being discovered from crustaceans that are phylogenetically distantly related to decapods (*e*.*g*., *E*. *superba*, *E*. *crystallorophias*) and from non-crustacean species (see Fig 6) [5,7,8,9,24,25,47,59], conclusions reached as summarized here could provide valuable information for understanding how functional diversification evolved within a peptide family.

## References

[1] Cooke IM, Sullivan RE (1982) Hormones and neurosecretion In: Atwood HL, Sandeman DC, editors. The Biology of Crustacea, Volume 3 New York: Academic Press pp. 206–290.

[2] Soyez D (1997) Occurrence and diversity of neuropeptides from the crustacean hyperglycemic hormone family in arthropods. Ann N Y Acad Sci 814: 319–323. 9160986 10.1111/j.1749-6632.1997.tb46174.x

[3] Kegel G, Reichwein B, Tensen CP, Keller R (1991) Amino acid sequence of crustacean hyperglycemic hormone (CHH) from the crayfish, *Orconectes limosus*: Emergence of a novel neuropeptide family. Peptides 12: 909–913. 1800954 10.1016/0196-9781(91)90036-o

[4] Keller R (1992) Crustacean neuropeptides: structures, functions, and comparative aspects. Experientia 48: 439–448. 1601108 10.1007/BF01928162

[5] Chen S-H, Lin C-Y, Kuo C-M (2005) *In silico* analysis of crustacean hyperglycemic hormone family. Mar Biotechnol 7: 193–206. 15933902 10.1007/s10126-004-0020-5

[6] De Kleijn DP, Van Herp F (1995) Molecular biology of neurohormone precursors in the eyestalk of Crustacea. Comp Biochem Physiol 112: 573–579. 10.1016/0305-0491(95)00126-3 8590372

[7] Christie AE (2008) Neuropeptide discovery in Ixodoidea: An *in silico* investigation using publicly accessible expressed sequence tags. Gen Comp Endocrinol 157: 174–185. 10.1016/j.ygcen.2008.03.027 18495123

[8] Christie AE, Nolan DH, Garcia ZA, McCoole MD, Harmon SM, Congdon-Jones B et al (2011) Bioinformatic prediction of arthropod/nematode-like peptides in non-arthropod, non-nematode members of the Ecdysozoa. Gen Comp Endocrinol 170: 480–486. 10.1016/j.ygcen.2010.11.002 21074533

[9] Montagné N, Desdevises Y, Soyez D, Toullec JY (2010) Molecular evolution of the crustacean hyperglycemic hormone family in ecdysozoans. BMC Evo Biol 10: 62. 10.1186/1471-2148-10-62 PMC2841656 20184761

[10] Santos EA, Keller R (1993) Crustacean hyperglycemic hormone (CHH) and the regulation of carbohydrate metabolism: current perspectives. Comp Biochem Physiol 106A: 405–411.

[11] Chang ES, Chang SA, Beltz BS, Kravitz EA (1999) Crustacean hyperglycemic hormone in the lobster nervous system: localization and release from cells in the subesophageal ganglion and thoracic second roots. J Comp Neurol 414: 50–56. 10494077

[12] Chang ES, Keller R, Chang SA (1998) Quantification of crustacean hyperglycemic hormone by ELISA in hemolymph of the lobster, *Homarus americanus*, following various stresses. Gen Comp Endocrinol 111: 359–66. 9707481 10.1006/gcen.1998.7120

[13] Lorenzon S, Edomi P, Giulianini PG, Mettulio R, Ferrero EA (2004) Variation of crustacean hyperglycemic hormone (cHH) level in the eyestalk and haemolymph of the shrimp *Palaemon elegans* following stress. J Exp Biol 207: 4205–4213. 15531641 10.1242/jeb.01264

[14] Webster SG (1996) Measurement of crustacean hyperglycemic hormone levels in the edible crab *Cancer pagurus* during emersion stress. J Exp Bio 199: 1579–1585. 9319482 10.1242/jeb.199.7.1579

[15] Zou H-S, Juan C-C, Chen S-C, Wang H-Y, Lee C-Y (2003) Dopaminergic regulation of crustacean hyperglycemic hormone and glucose levels in the hemolymph of the crayfish *Procambarus clarkii* . J Exp Zool 298: 44–52. 10.1002/jez.a.10273 12840838

[16] Chang C-C, Tsai T-W, Hsiao N-W, Chang C-Y, Lin C-L, Watson RD et al (2010) Structural and functional comparisons and production of recombinant crustacean hyperglycemic hormone (CHH) and CHH-like peptides from the mud crab *Scylla olivacea* . Gen Comp Endocrinol 167: 68–76. 10.1016/j.ygcen.2010.02.013 20171218

[17] Chang ES, Prestwich GD, Bruce MJ (1990) Amino acid sequence of a peptide with both molt-inhibiting activity and hyperglycemic activities in the lobster *Homarus americanus* . Biochem Biophys Res Commun 171: 818–826. 2169734 10.1016/0006-291x(90)91219-i

[18] Chung JS, Dircksen H, Webster SG (1999) A remarkable, precisely timed release of hyperglycemic hormone from endocrine cells in the gut is associated with ecdysis in the crab *Carcinus maenas* . Proc Natl Acad Sci USA 96: 13103–13107. 10557280 10.1073/pnas.96.23.13103 PMC23907

[19] Dircksen H, Böcking D, Heyn U, Mandel C, Chung JS, Baggerman G et al (2001) Crustacean hyperglycemic hormone (CHH)-like peptides and CHH-precursor-related peptides from pericardial organ neurosecretory cells in the shore crab, *Carcinus maenas*, are putatively spliced and modified products of multiple genes. Biochem. J. 356: 159–170. 11336648 10.1042/0264-6021:3560159 PMC1221824

[20] Khayat M, Yang W, Aida K, Nagasawa H, Tietz A, Funkenstein B et al (1998) Hyperglycemic hormones inhibit protein and mRNA synthesis in *in vitro*-incubated ovarian fragments of the marine shrimp *Penaeus semisulcatus* . Gen Comp Endocrinol 110: 307–318. 9593651 10.1006/gcen.1998.7078

[21] Serrano L, Blanvillain G, Soyez D, Charmantier G, Grousset E, Aujoulat F et al (2003) Putative involvement of crustacean hyperglycemic hormone isoforms in the neuroendocrine mediation of osmoregulation in the crayfish *Astacus leptodactylus* . J Exp Biol 206: 979–988. 12582140 10.1242/jeb.00178

[22] Spanings-Pierrot C, Soyez D, Van Herp F, Gompel M, Skaret G, Grousset E et al (2000) Involvement of crustacean hyperglycemic hormone in the control of gill ion transport in the crab *Pachygrapsus marmoratus* . Gen Comp Endocrinol 119: 340–350. 11017781 10.1006/gcen.2000.7527

[23] Yasuda A, Yasuda Y, Fujita T, Naya Y (1994) Characterization of crustacean hyperglycemic hormone from the crayfish (*Procambarus clarkii*): Multiplicity of molecular forms by stereoinversion and diverse functions. Gen Comp Endocrinol 95: 387–398. 7821776 10.1006/gcen.1994.1138

[24] Kung P-C, Wu S-H, Nagaraju GPC, Tsai W-S, Lee C-Y (2013). Crustacean hyperglycemic hormone precursor transcripts in the hemocytes of the crayfish *Procambarus clarkii*: novel sequence characteristics relating to gene splicing pattern and transcript stability. Gen Comp Endocrinol 186: 80–85. 10.1016/j.ygcen.2013.03.010 23518482

[25] Wu S-H, Chen Y-J, Huang S-Y, Tsai W-S, Wu H-J, Hsu T-T et al (2012) Demonstration of expression of a neuropeptide-encoding gene in crustacean hemocytes. Comp Biochem Physiol 161A: 463–468. 10.1016/j.cbpa.2012.01.007 22269107

[26] Wanlem S, Supamattaya K, Tantikitti C, Prasertsan P, Graidist P (2011) Expression and applications of recombinant crustacean hyperglycemic hormone from eyestalks of white shrimp (*Litopenaeus vannamei)* against bacterial infection. Fish Shellfish Immunol 30: 877–885. 10.1016/j.fsi.2011.01.014 21272649

[27] Katayama H, Ohira T, Aida K, Nagasawa H (2002) Significance of a carboxyl-terminal amide moiety in the folding and biological activity of crustacean hyperglycemic hormone. Peptides 23: 1537–1546. 12217413 10.1016/s0196-9781(02)00094-3

[28] Marco HG, Brandt W, Stoeva S, Voelter W, Gäde G (2000) Primary structures of a second hyperglycemic peptide and of two truncated forms in the spiny lobster, *Jasus lalandii* . Peptides 21: 19–27. 10704715 10.1016/s0196-9781(99)00171-0

[29] Mosco A, Edomi P, Guarnaccia C, Lorenzon S, Pongor S, Ferrero EA et al (2008) Functional aspects of cHH C-terminal amidation in crayfish species. Regul Pept 147: 88–95. 10.1016/j.regpep.2008.01.005 18281112

[30] Lacombe C, Greve P, Martin G (1999) Overview on the sub-grouping of the crustacean hyperglycemic hormone family. Neuropeptides 33: 71–80. 10657474 10.1054/npep.1999.0016

[31] Lee C-Y, Tsai K-W, Tsai W-S, Jiang J-Y, Chen Y-J (2014) Crustacean hyperglycemic hormone: structural variants, physiological functions, and cellular mechanism of action. J Mar Sci Technol 22: 75–81.

[32] Webster SG, Keller R, Dircksen H (2012) The CHH-superfamily of multifunctional peptide hormones controlling crustacean metabolism, osmoregulation, moulting, and reproduction. Gen Comp Endocrinol 175: 217–233. 10.1016/j.ygcen.2011.11.035 22146796

[33] Katayama H, Ohira T, Nagata S, Nagasawa H (2004) Structure-activity relationship of crustacean molt-inhibiting hormone from the Kuruma prawn *Marsupenaeus japonicas* . Biochemistry 43: 9629–9635. 15274617 10.1021/bi049433v

[34] Wang Y-J, Zhao Y, Meredith J, Phillips JE, Theilmann DA, Brock HW (2000) Mutational analysis of the C-terminus in ion-transport peptide (ITP) expressed in *Drosophila* Kc1 cells. Arch Insect Biochem Physiol 45: 129–138. 11169752 10.1002/1520-6327(200011)45:3<129::AID-ARCH4>3.0.CO;2-L

[35] Zhao Y, Meredith J, Brock HW, Phillips JE (2005) Mutational analysis of the N-terminus in *Schistocerca gregaria* ion-transport peptide expressed in *Drosophila* Kc1 cells. Arch Insect Biochem Physiol 58: 27–38. 15599935 10.1002/arch.20028

[36] Katayama H, Nagata K, Ohira T, Yumoto F, Tanokura M, Nagasawa H (2003) The solution structure of molt-inhibiting hormone from the Kuruma prawn *Marsupenaeus japonicus* . J Biol Chem 278: 9620–9623. 12519766 10.1074/jbc.M212962200

[37] Mettulio R, Giulianini PG, Ferrero EA, Lorenzon S, Edomi P (2004) Functional analysis of crustacean hyperglycemic hormone by in vivo assay with wild-type and mutant recombinant proteins. Regul Pept 119: 189–197. 15120480 10.1016/j.regpep.2004.02.002

[38] Drach P, Tchernigovtzeff C (1967) Sur la méthode de détermination des stades ďintermue et son application générale aux Crustacés. Vie Milieu A 18: 595–609.

[39] Schagger H, Von Jagow G (1987) Tricine-sodium dodecyl sulfate-polyacrylamide gel electrophoresis for the separation of proteins in the range from 1 to 100 kDa. Anal Biochem 166: 368–379. 2449095 10.1016/0003-2697(87)90587-2

[40] Hsiao N-W, Samuel D, Liu Y-N, Chen L-C, Yang T-Y, Jayaraman G et al (2003) Mutagenesis Study on the Zebra Fish SOX9 High-Mobility Group: Comparison of Sequence and Non-Sequence Specific HMG Domains. Biochemistry 42: 11183–11193. 14503868 10.1021/bi034678d

[41] Tsai K-W, Chang S-G, Wu H-J, Shih H-Y, Chen C-H, Lee C-Y (2008) Molecular cloning and differential expression pattern of two structural variants of the crustacean hyperglycemic hormone family from the mud crab *Scylla olivacea* . Gen Comp Endocrinol 159, 16–25. 10.1016/j.ygcen.2008.07.014 18713635

[42] Ohira T, Tsutsui N, Nagasawa H, Wilder MN (2006). Preparation of two recombinant crustacean hyperglycemic hormones from the giant freshwater prawn, *Macrobrachium rosenbergii*, and their hyperglycemic activities. Zool Sci 23: 383–391. 16702772 10.2108/zsj.23.383

[43] Aguilar MB, Soyez D, Falchetto R, Arnott D, Shabanowitz J, Hunt DF et al (1995) Amino acid sequence of the minor isomorph of the hyperglycemic hormone (CHH-II) of the Mexican crayfish *Procambarus bouvieri* (Ortmann): presence of a D amino acid. Peptides 16: 1375–1383. 8745046 10.1016/0196-9781(95)02024-1

[44] Bulau P, Meisen I, Reichwein-Roderburg B, Peter-Katalinic J, Keller R (2003) Two genetic variants of the crustacean hyperglycemic hormone (CHH) from the Australian crayfish, *Cherax destructor*: detection of chiral isoforms due to posttranslational modification. Peptides 24: 1871–1879. 15127939 10.1016/j.peptides.2003.10.002

[45] Soyez D, Laverdure AM, Kallen J, Van Herp F (1998) Demonstration of a cell-specific isomerization of invertebrate neuropeptides. Neuroscience 82: 935–942. 9483547 10.1016/s0306-4522(97)00254-6

[46] Soyez D, Van Herp F, Rossier J, LeCaer JP, Tensen CP, Lafont R. (1994) Evidence for a conformational polymorphism of invertebrate neurohormones. J Biol Chem 269: 18295–18298. 8034574

[47] Wu H-J, Tsai W-S, Huang S-Y, Chen Y-J, Chen Y-H, Hsieh Y-R et al (2012) Identification of crustacean hyperglycemic hormone (CHH) and CHH-like (CHH-L) peptides in the crayfish *Procambarus clarkii* and localization of functionally important regions of CHH. Zool. Stud. 51: 288–297.

[48] Lebaupain F, Boscameric M, Pilet E, Soyez D, Kamech N. 2012 Natural and synthetic isoforms of crustacean hyperglycemic hormone from the crayfish *Astacus leptodactylus*: Hyperglycemic activity and hemolymphatic clearance. Peptides 34: 65–73. 10.1016/j.peptides.2012.01.019 22314080

[49] Mosco A, Zlatev V, Guarnaccia C, Pongor S, Campanella A, Zahariev S et al (2012) Novel protocol for the chemical synthesis of crustacean hyperglycemic hormone analogues–An efficient experimental tool for studying their functions. Plos One 7: 1–9, e30052. 10.1371/journal.pone.0030052 PMC3256185 22253873

[50] Mosco A, Zlatev V, Guarnaccia C, Giulianini PG (2015) Functional analysis of a mutated analogue of the crustacean hyperglycaemic hormone from the crayfish Pontastacus leptodactylus. See comment in PubMed Commons belowJ Exp Zool A Ecol Genet Physiol. 323:121–127. 10.1002/jez.1909 25678476

[51] Chung SJ, Zmora N, 2008 Functional studies of crustacean hyperglycemic hormones (CHHs) of the blue crab, *Callinectes sapidus*—the expression and release of CHH in eyestalk and pericardial organ in response to environmental stress. FEBS J. 275, 693–704. 10.1111/j.1742-4658.2007.06231.x 18190527

[52] Chen SH, Lin CY, Kuo CM (2004) Cloning of two crustacean hyperglycemic hormone isoforms in freshwater giant prawn (*Macrobrachium rosenbergii*): evidence of alternative splicing. Mar. Biotechnol. 6, 83–94. 14583813 10.1007/s10126-003-0014-8

[53] Toullec J-Y, Serrano L, Lopez P, Soyez D, Spanings-Pierrot C (2006) The crustacean hyperglycemic hormones from an euryhaline crab *Pachygrapsus marmoratus* and a fresh water crab *Potamon ibericum*: eyestalk and pericardial isoforms. Peptides 27: 1269–1280. 16413086 10.1016/j.peptides.2005.12.001

[54] Nagai C, Asazuma H, Nagata S, Nagasawa H (2009) Identification of a second messenger of crustacean hyperglycemic hormone signaling pathway in the kuruma prawn *Marsupenaeus japonicus* . Ann NY Acad Sci 1163: 478–480. 10.1111/j.1749-6632.2009.04452.x 19456392

[55] Yang WJ, Aida K, Nagasawa H (1997) Amino acid sequences and activities of multiple hyperglycemic hormones from the kuruma prawn, *Penaeus japonicus* . Peptides 18: 479–485. 9210164 10.1016/s0196-9781(96)00332-4

[56] Chan S-M, Gu P-L, Chu K-H, Tobe SS (2003) Crustacean neuropeptide genes of the CHH/MIH/GIH family: implications from molecular studies. Gen Comp Endocrinol 134: 214–219. 14636627 10.1016/s0016-6480(03)00263-6

[57] Meredith J, Ring M, Macins A, Marschall J, Cheng NN, Theilmann D et al (1996) Locust ion transport peptide (ITP): Primary structure, cDNA and expression in a baculovirus system. J Exp Biol 199: 1053–1061. 8786332 10.1242/jeb.199.5.1053

[58] Phillips JE, Meredith J, Adusley N, Ring M, Macins A, Brock HW (2001) Ion transport peptide (ITP): structure, function, evolution In: Goos H.J., Rastogi R.K., Vaudry H., Pierantoni R., editors. Perspectives in comparative endocrinology: Unity and diversity. Bologna: Medimond pp. 745–752.

[59] McCowan G, Garb JE (2014) Recruitment and diversification of an ecdysozoan family hormones for black widow spider venom expression. Gene 536: 366–375. 10.1016/j.gene.2013.11.054 24316130 PMC4172349

